# Application of Whole Genome Resequencing in Mapping of a Tomato Yellow Leaf Curl Virus Resistance Gene

**DOI:** 10.1038/s41598-018-27925-w

**Published:** 2018-06-25

**Authors:** Yinlei Wang, Jing Jiang, Liping Zhao, Rong Zhou, Wengui Yu, Tongmin Zhao

**Affiliations:** 1 Institute of Vegetable Crop, Jiangsu Academy of Agricultural Science, Nanjing, Jiangsu China; 2 Laboratory for Genetic Improvement of High Efficiency Horticultural Crops in Jiangsu province, Jiangsu Nanjing, China

## Abstract

Tomato yellow leaf curl virus (TYLCV) has significantly impacted the tomato industry around the world, and the use of insecticides and insect nets have not effectively controlled the spread of this pathogen. The tomato line AVTO1227 is highly resistant to TYLCV. In this study, F_2_ and BC_1_ populations derived from AVTO1227 and the susceptible line Money maker were used to assess the genetic mechanism underlying TYLCV resistance. We have identified a recessive TYLCV resistance gene, hereby designated as *ty-5*, which is linked to *SlNACI*. Genomic DNA pools from resistant and susceptible groups were constructed, and their genomes were resequenced. The *ty-5* gene was identified on an interval encompassing the genomic positions 2.22 Mb to 3.19 Mb on tomato chromosome 4. Genotyping using linkage markers further mapped *ty-5* within the interval between markers ty5–25 and ty5–29, where only the *pelota* gene is located. Consequently, *pelota* was considered as the candidate gene corresponding to *ty-5*. Two nucleotide transversions within the promoter region and one transversion in exon region of the *pelota* gene were detected in the parental lines. However, the relative transcript levels of *pelota* did not significantly differ among the three tomato lines, regardless of TYLCV infection. This study will facilitate marker-assisted breeding for resistance to TYLCV and lay a foundation for the research of the resistance mechanism of *ty-5* in tomato.

## Introduction

Tomato yellow leaf curl disease (TYLCD) is caused by tomato yellow leaf curl virus (TYLCV) and is one of the most important diseases of tomato^1^. TYLCV belongs to the genus *Begomovirus* within the family *Geminiviridae*. The whitefly, *Bemisia tabaci*, transmits TYLCV from diseased plants to healthy plants^2,3^, and the host range in plants is very wide, which include various types of vegetables, flowers, and weeds^4^. Tomatoes that are infected with TYLCV display leaf curling, yellowing, and stunting of plant growth. Severe damage due to TYLCV result in plant death and subsequently no crop yield^5^, ultimately leading to substantial economic losses. In 2006, TYLCD outbreak was reported in many areas of China, resulting in a significant decrease in tomato production^6,7^. The primary strategies for controlling TYLCV transmission involve controlling populations of the whitefly vector and choosing tomato cultivars that are resistant to the virus. However, it is generally difficult to control whiteflies as these have strong reproductive and migratory behaviors. Furthermore, previous applications of large quantities of insecticides may have led to whitefly resistance to insecticides^8,9^. Consequently, planting of cultivars that are more resistant to TYLCV infection has become a more prominent strategy to maintain tomato production^10^.

At the onset of a TYLCV outbreak, genes related to resistance were not present in cultivated tomato strains. However, several wild tomato species exhibited high levels of resistance to TYLCV^11^, and six major TYLCV resistance genes have been discovered in wild species. The *Ty-1* and *Ty-3* alleles were introgressed into tomato via *Solanum chilense* accessions LA1969 and LA2779, respectively. These alleles map to chromosome 6 and encode an RNA-dependent RNA polymerase^12,13^. *Ty-*2 originated from *S. habrochiates* strain B6013 and is located on the long arm of chromosome 11^14,15^. *Ty-4* is located on the long arm of chromosome 3 and was introgressed from *S. chilense* strain LA1932^16^. Marker SlNAC1 is linked to *ty-5*^17,18^ and was derived from a *S. peruvianum* accession. Lastly, *Ty-6* is a novel resistance gene that is located on chromosome 10 and was also derived from *S. chilense* strain LA2779^19^.

Of the above resistance genes, *Ty-1*, *Ty-2*, and *Ty-3* have been primarily introgressed into hybrid tomato cultivars in China and have prevented substantial TYLCV-related losses in the tomato industry. However, the virus exhibits a very high mutation rate, and the introgression of other resistance genes into hybrid cultivars is necessary^20^. The TYLCV resistant line AVTO1227 was introduced from the World Vegetable Center in 2013. AVTO1227 is highly resistant to TYLCV after inoculation with whiteflies that are viruliferous for the TYLCV strain. Genotypic analysis using the marker SlNAC1 indicated that their resistance is conferred by *ty-5*. Consequently, developing tomato cultivars with *ty-5* is of great interest for tomato breeding programs of China. In particular, fine-mapping of *ty-5* may substantially aid these efforts.

Recently, whole genome resequencing (WGR) has been widely adopted in gene mapping^21,22^. WGR is faster and more efficient in developing linkage markers compared to traditional methods. In this study, we conducted WGR of two genomic DNA pools representing tomatoes that are resistant and susceptible to TYLCV and were developed from plants in a TYLCV-inoculated F_2_ population. Polymorphic SNPs and INDELs were then identified between the two genome pools. The region of the chromosome where the TYLCV resistance gene was located was further analyzed. Combined genotypic and phenotypic analyses of the F_2_ population indicated that *pelota* is the gene corresponding to *ty-5*. Finally, two transversions within this region were detected.

## Results

### Comparison of tomato line phenotypes after TYLCV inoculation

**Figure 1:**
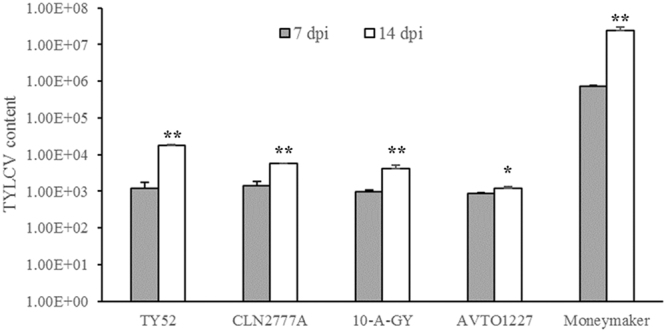
TYLCV abundances in tomato lines with different genotypes 7 and 14 days post inoculation with an infectious TYLCV-IL clone. TYLCV abundances are based on RT-PCR analysis of gene copies. The line designations TY52, CLN2777A, 10-A-GY, AVTO1227, and Money maker refer to those with the *Ty-1*, *Ty-2*, *Ty-3*, and *ty-5* resistance genes and no resistance genes, respectively. Asterisks above the bars represent significant differences between 7 and 14 days post inoculation. (*P < 0.05, **P < 0.01).

Tomato lines with different levels of TYLCV resistance were inoculated, and the severity of disease symptoms was subsequently evaluated. Two inoculation methods were used to evaluate disease severity: whiteflies viruliferous for the TYLCV-IL strain and *Agrobacterium* carrying the infectious TYLCV-IL clone (Table 1). In contrast to the high disease severity index (DSI) results for the control tomato line Money maker that was susceptible to infection, all the other lines were highly resistant to TYLCV using both inoculation methods. The CLN2777A and AVTO1227 lines did not display any typical TYLCV disease symptoms, although PCR analysis indicated that all five tomato lines carried the TYLCV. Quantitative RT-PCR analysis also confirmed that these lines harbored the TYLCV. Comparison of TYLCV levels at 7 and 14 day post inoculation (dpi) indicated significant differences. The observed increase in TYLCV content in material 1227 was not as high as that in the other materials. TYLCV content in Money maker was significantly higher compared to the other lines regardless of the number of days after inoculation (7 or 14 dpi) (Fig. 1).

**Table 1:** Tomato yellow leaf curl virus disease severity index values for five inbred tomato lines with two inoculation methods.

Tomato line	Resistance gene	Disease severity index (DSI)	
		Whiteflies	*Agrobacterium*
Money maker	Control	4.0^A^ ± 0.0 (n = 30)	4.0^A^ ± 0.0 (n = 30)
TY52	*Ty-1*	0.8^B^ ± 0.1 (n = 30)	0.0^B^ ± 0.0 (n = 30)
CLN2777A	*Ty-2*	0.0^D^ ± 0.0 (n = 30)	0.0^B^ ± 0.0 (n = 30)
10-A-GY	*Ty-3a*	0.5^C^ ± 0.1 (n = 30)	0.0^B^ ± 0.0 (n = 30)
AVTO1227	*ty-5*	0.0^D^ ± 0.0 (n = 30)	0.0^B^ ± 0.0 (n = 30)

The results are displayed as the mean ± SE; n = number of plants for each group; Superscript letters represent statistically significant differences at *P* < 0.05 based on Duncan’s multiple range test.

### Inheritance of TYLCV resistance in line AVTO1227

AVTO1227 (P1) and Money maker (P2) were used to develop the F_1_, F_2_, BC_1_P_1_ and BC_1_P_2_ populations. Population phenotypes were then evaluated and recorded 45 days following TYLCV inoculation. All F_1_ and BC_1_P_2_ seedlings showed stunting and yellowing symptoms. In contrast, F_2_ and BC_1_P_1_ seedlings infected with TYLCV exhibited variations in symptoms (Table 2). The segregation ratio of resistant-susceptible individuals indicated that a single recessive gene conferred resistance to TYLCV in AVTO1227. Analysis of the molecular marker SlNACI then indicated that the candidate recessive gene in AVTO1227 is *ty-5*.

**Table 2:** Variation in resistance to TYLCV in different generations.

Generation	Number of plants			Actual ratio of R:S	Theoretical ratio of R:S	*X* ^*2*^
	Total	Resistant	Susceptible			
AVTO1227	30	30	0	/		/
Money maker	30	0	30	/		/
F_1_	30	0	30	/		/
F_2_	2136	538	1598	1:2.97	1:3	0.0306
BC_1_P_1_	38	17	21	1:1.23	1:1	0.2368
BC_1_P_2_	41	0	41	/		/

$${{\rm{X}}}_{0.05}^{2}$$ = 3.84.

### BSA-seq analysis

**Figure 2:**
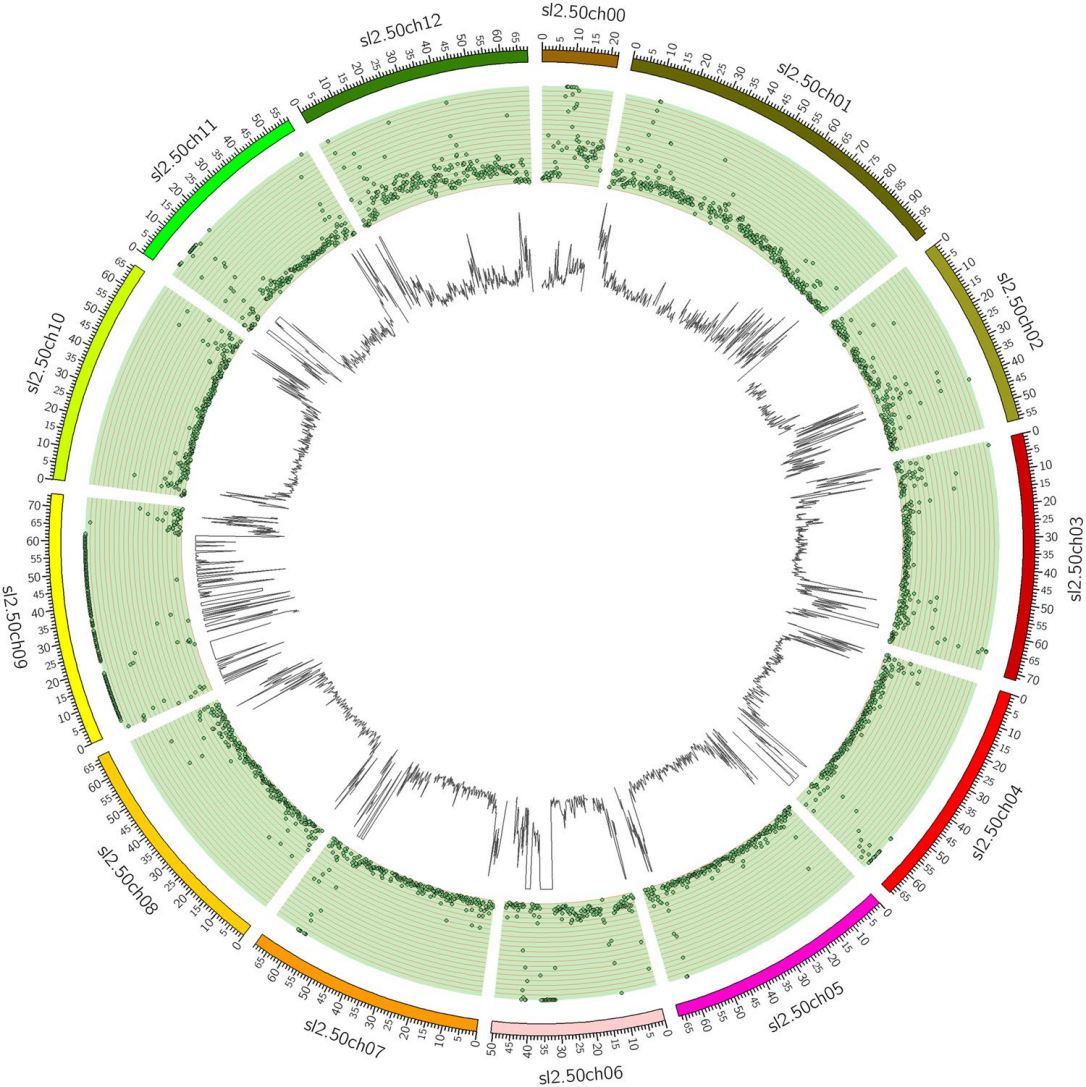
Distribution of polymorphic variations between resistant and susceptible tomato genomes. The external ring is the reference genome. The middle ring shows the distribution of SNPs as indicated by a scatter plot. The inner ring is the distribution of INDELs as indicated by a linear histogram.

Two DNA genome pools, namely, resistant (R-) and susceptible (S-), were constructed for BSA-seq analysis using Illumina high-throughput sequencing. A total of 253,249,960 and 239,118,590 clean reads were obtained from the R and S-pools, respectively. Raw data were deposited in the NCBI Sequence Read Archive under the accession number PRJNA312569. About 91.7% of these clean reads were mapped onto the tomato genome, and resulted in 96.66% and 96.86% coverage, with at least 10× depth in the R- and S-pools, respectively. A total of 1,709,042 SNPs and 94,066 INDELs were identified as differential between the R- and S-pools. Circos software was used to analyze the distribution of the polymorphisms, which indicated that the distribution of SNPs and INDELs across chromosomes is not uniform (Fig. 2). For example, there were only 1,983 differential INDELs on chromosome 5, whereas there were 46,592 differential INDELs on chromosome 9.

### Gene location association analysis using BSA-seq

**Figure 3:**
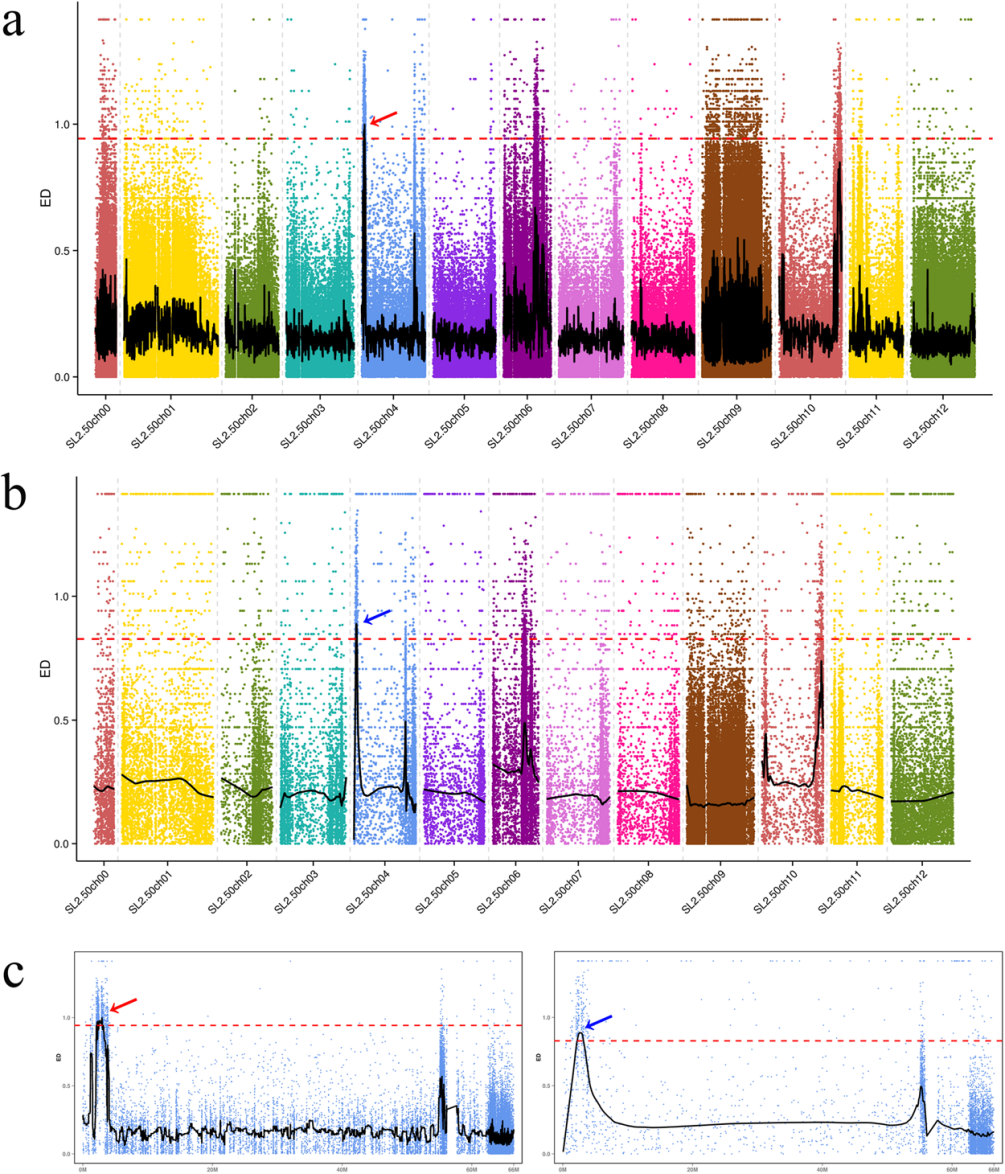
Euclidean distance association analysis of the *ty-5* candidate interval in the tomato genome. (**a**) ED calculated based on the SNP-index. The red arrow indicates the chromosomal location of the *ty-5*. (**b**) ED calculated based on the INDEL-index. The blue arrow indicates the chromosomal location of the *ty-5*.(**c**) Enlarged view of chromosome 4 from Fig. 3a (left) and 3b (right). Different colors in Fig. 3a and b represent different tomato chromosomes. The red dotted line indicates a 0.5% empirical outlier threshold for the BSA sequencing.

The Δ(SNP_index), Δ(INDEL_index), and ED values were calculated to conduct association mapping. Peak regions above the threshold value are defined as regions where *ty-5* may be located. SNP analysis indicated that the region encompassing the genomic positions 2,084,876–3,198,109 on chromosome 4 may be the candidate location of *ty-5*. Concordantly, INDEL analysis suggested that the interval encompassing the genomic positions 2,227,907–3,198,109 region on chromosome 4 was identified as candidate region of *ty-5*. Combining both results, the TYLCV resistance gene *ty-5* was mapped to the interval encompassing the genomic positions 2,227,907–3,198,050 region on tomato chromosome 4 (Fig. 3). A total of 129 genes are located in this 970-kb region. Some of these genes contain NB-ARC domains, and functional annotation suggests that these play a role in disease resistance. The PCR product of the linkage marker SlNACI was then sequenced, and sequence comparison indicated that this marker is located within the *ty-5* interval region.

### Mapping of ty-5 gene

The 970-kb interval harbored 940 SNPs and 184 INDELs between the two experimental groups. One SNP was identified in nearly every kilobase and polymorphisms between parents can be used in *ty-5* mapping. DNA samples from 10 TYLCV-resistant and 10 TYLCV-susceptible plants were used to determine linkage of markers to resistance genes. The genotypes of 2,136 F_2_ plants were assessed using the SlNACI marker and ty5–17. The resistance gene was identified in the interval between these two markers, and 64 recombinant plants were detected between them. Fine-mapping was conducted to better resolve the location of *ty-5*, and linkage markers between SlNACI and ty5–17 were used in genotyping (Table 3). Twenty-five recombinants were ultimately identified between ty5–13 and ty5–17, narrowing down the region harboring the resistance gene to an interval of 101 kb.

**Table 3:** Molecular marker information for mapping the *ty-5* gene.

Marker	Primer sequence (5′-3′, F/R)	Genomic position	Marker type	R.E^a^
*SlNACI*	TGCCTGGTTTCTGCTGTCA TAAAGCTGAAGAAGGACTTACCCT	2,857,095	CAPS	*Taq* I
ty5-1	ATACAAGGTACACCAACA GTGAGCCATTTTCTAACG	2,862,405	CAPS	*Hinf* I
ty5-5	CCTGAAGTCCACAACCCTT CTCGGACGTGCGTTCTAT	2,989,381	CAPS	*BstU I*
ty5–13	CAAGCAAGTAAGCCAAAG AACCAAAGTCGCTAAAGG	3,074,561	INDEL	None
ty5–17	TAACAAAGCCCTCAAAGC GTCTCCGAAACGTAATCC	3,176,189	INDEL	None
ty5–25	GTAATCGTGTCATTTCGG TTTGAGTTTCAATGTAGGG	3130934	SNP	None
ty5–26	GTATAGTGTTTTAGACGATGCA TGGAGAAAATAAAAGTGGC	3,105,620	SNP	None
ty5–29	GAAAACCTCCACTACTTGAA TGCCGTCTTAGTAAACCA	3,116,418	SNP	None

^a^R.E. restriction endonuclease used to obtain polymorphism.

**Figure 4:**
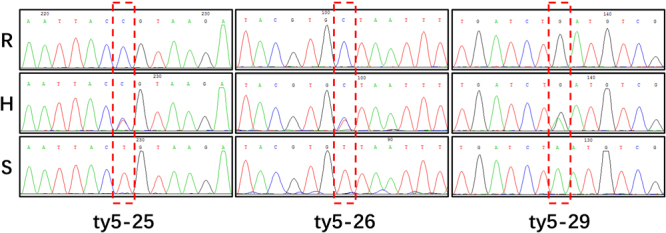
Identification of SNP polymorphisms via PCR product sequencing. Red boxes indicate identified polymorphisms. Groups are labeled such that R indicates the homozygous resistant genotype, H indicates the heterozygous genotype, and S indicates the homozygous susceptible genotype.

**Figure 5:**
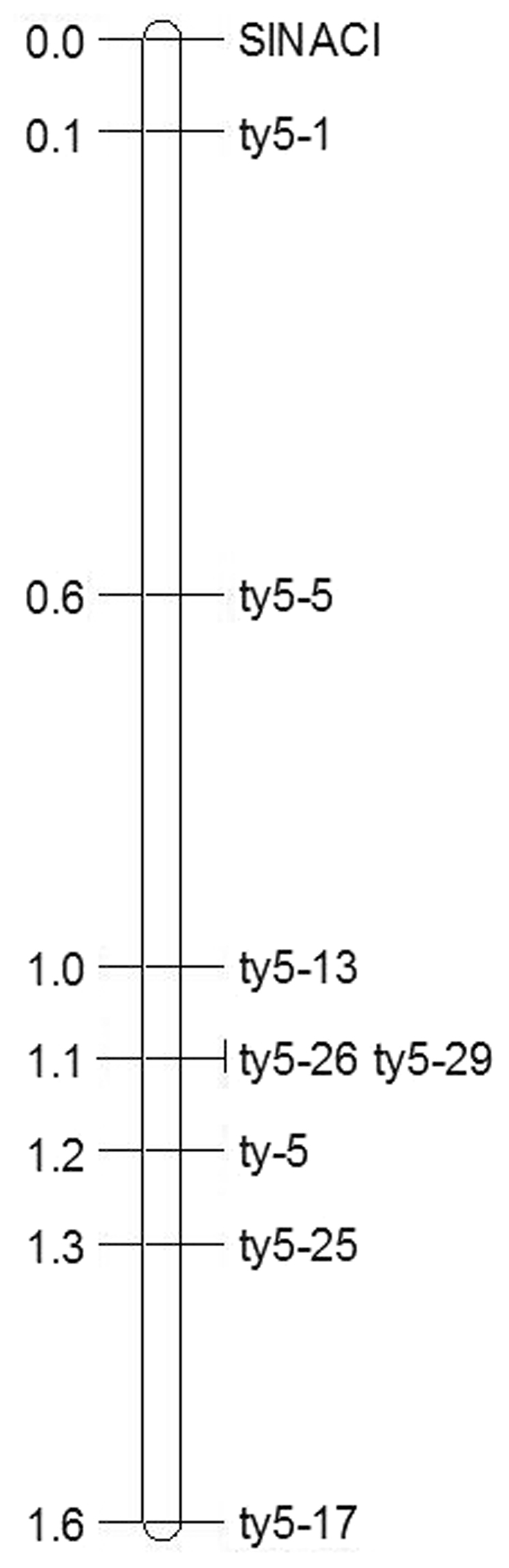
Genetic map of the TYLCV resistance locus *ty-5* on tomato chromosome 4. Linkage maps were constructed from F_2_ populations that were derived from a cross between the susceptible tomato line Money maker and the resistant line AVTO1227. Numbers on the left of the vertical line indicate genetic distances (in centimorgans).

Genotyping did not identify a content marker that could be directly used in electrophoretic analysis. Consequently, SNP polymorphisms were further assessed by sequencing PCR products. Three sequence markers, ty5–25, ty5–26, and ty5–29 were used to analyze the 25 recombinants (Fig. 4). All 25 recombinants with ty5–26 and ty5–29 exhibited the same genotype (Table 4). Seven of these plants indicated that recombination occurred between ty5–25 and ty5–29. The F_2_ phenotype of these plants then indicated that the *ty-5* gene is located within the chromosomal interval between ty5–25 and ty5–13. Three of the seven recombinant plants, namely, 45, 1198, and 1683, were selfed and received F_3_ seeds. The three F_3_ populations were inoculated with TYLCV, and disease severity was then analyzed. All individuals of the F_3_ population of plant 1683 exhibited TYLCV resistance, which coincides with the genotype of the F_2_ progeny. In contrast, all individuals of the F_3_ population from plant 45 showed susceptibility to TYLCV. The F_3_ population from plant 1198 exhibited variations in TYLCV resistance. Order of Markers on the chromosome 4 is from SlNACI to ty5–17, just as that showed in the first row of Table 4. All these results indicate that *ty-5* is located between markers ty5–25 and ty5–29 (Fig. 5), which are located on chromosome 4, between genomic positions 3116418 of ty5–29 and 3,130,934 of ty5–25. Over this 14.5 kbp genomic interval, only one gene, *pelota*, is present. The *pelota* gene, which corresponds to *ty-5*, confers TYLCV resistance in AVTO1227. In the interval harboring the *pelota* gene, two transversions within the promoter region and a SNP in the exon were detected. The transversion A-to-C in the first exon of *pelota* resulting in a Valine16-to-Glycine substitution in AVTO1227 (Table 5).

**Table 4:** Genotype and phenotype analysis of plants that underwent recombination between ty5–13 and ty5–17.

F_2_ plant#	SlNACI	ty5–1	ty5-5	ty5-13	ty5-26	ty5–29	ty5–25	ty5–17	PT-F2^b^	PT-F_3_
1077	A^a^	A	A	A	H	H	H	H	S	
1439	A	A	A	A	H	H	H	H	S	
1683	A	A	A	A	A	A	H	H	R	R
681	B	B	B	B	B	B	H	H	S	
856	B	B	B	B	B	B	B	H	S	
864	B	B	B	B	B	B	B	H	S	
1198	B	B	B	B	B	B	H	H	S	R/S
1220	B	B	B	B	B	B	B	H	S	
1331	B	B	B	B	B	B	H	H	S	
1345	B	B	B	B	B	B	B	H	S	
1510	B	B	B	B	B	B	H	H	S	
2081	B	B	B	B	B	B	B	H	S	
600	H	H	H	H	H	H	A	A	S	
45	H	H	H	H	H	H	B	B	S	S
151	H	H	H	H	H	H	H	B	S	
859	H	H	H	H	A	A	A	A	R	
925	H	H	H	H	B	B	B	B	S	
979	H	H	H	H	H	H	H	A	S	
1237	H	H	H	H	H	H	H	B	S	
1470	H	H	H	H	H	H	H	A	S	
1589	H	H	H	H	B	B	B	B	S	
1776	H	H	H	H	H	H	H	A	S	
1885	H	H	H	H	H	H	H	B	S	
2059	H	H	H	H	A	A	A	A	R	
2123	H	H	H	H	H	H	H	A	S	

^a^Genotype designation: (A) homozygous resistant locus, (B) homozygous susceptible, (H) heterozygous.

^b^PT: phenotype of the F_2_ or F_3_ population after inoculation with TYLCV. Phenotype designations: (R) F_2_ or F_3_ plants showing resistance to TYLCV. (S) F_2_ or F_3_ populations showing susceptibility to TYLCV. (R/S) indicates that the F_3_ population phenotype varied after inoculation with TYLCV.

**Table 5:** Sequence difference of R- and S-pool within the interval between markers ty5–25 and ty5–29.

position	Ref^a^	Alt^b^	R-pool		S-pool		Gene name or effect
			Read depth of ref	Read depth of alt	Read depth of ref	Read depth of alt	
3116418	A	G	0	45	27	11	intergenic
3125501	A	C	0	39	21	6	*pelota*
3125924	T	A	21	0	6	13	upstream of *pelota*
3125925	A	T	0	21	13	6	upstream of *pelota*
3127885	C	T	0	31	26	11	intergenic
3127973	T	C	0	47	30	18	intergenic
3128931	T	C	0	25	12	8	intergenic
3129254	G	T	0	38	15	6	intergenic
3130934	T	C	0	27	16	6	intergenic

^a^Ref: Reference sequence. ^b^Alt: Alternate sequence.

### Expression of *ty-5* under different conditions

**Figure 6:**
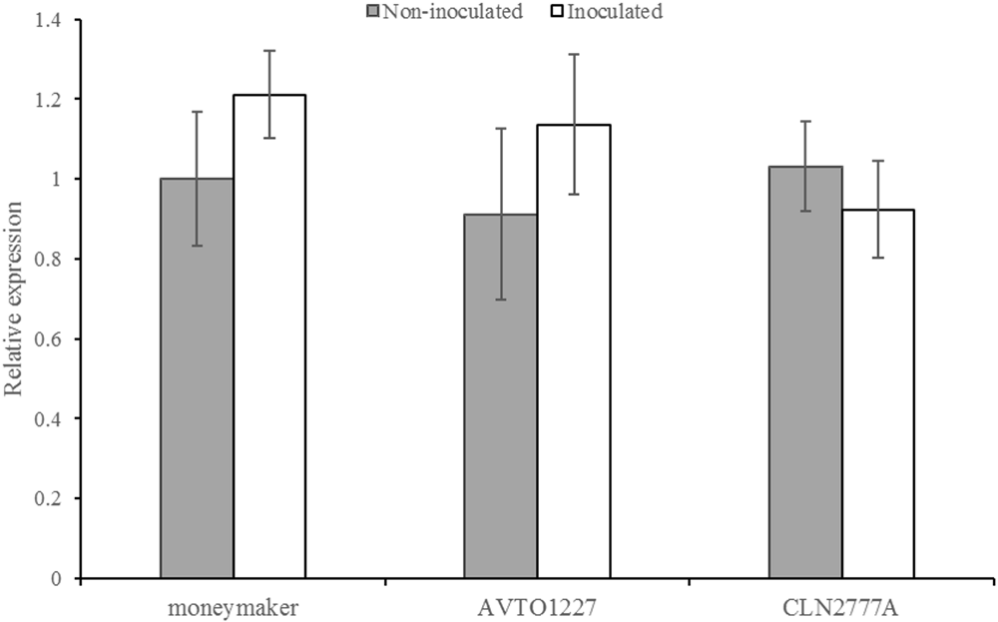
Relative expression of *ty-5* transcripts based on real-time RT-PCR analysis in three tomato lines seven days after agroinfiltration. The *GAPDH* gene was used as internal reference. Error bars represent standard errors of three biological replicates. Significant differences were analyzed at the 0.05 level.

Three tomato lines, namely, AVTO1227, Money maker, and CLN2777A, were inoculated with TYLCV. Seven days post-inoculation, inoculated and uninoculated control samples were collected for assessment of *ty-5* expression levels. Quantitative RT-PCR (qPCR) analysis indicated that *ty-5* and its allele, *Ty-5*, were expressed in all samples in both experimental groups. Furthermore, the expression level of these alleles did not significantly differ among all six samples (*P* = 0.05, Fig. 6).

## Discussion

Since 2006, TYLCV has emerged in the majority of tomato-producing areas in China^6^. At that time, almost all of the primary cultivated varieties of tomatoes were susceptible to TYLCV. However, since then, several tomato varieties with TYLCV resistance have been bred. In particular, *Ty-1*, *Ty-2*, and *Ty-3* have been widely used in tomato cultivars to confer resistance. However, long-term use of these genes may lead to the development of new mechanisms by which the pathogen can overcome resistance. Consequently, new TYLCV resistance genes are needed, and one such gene, *ty-5*, has been shown to confer superior resistance, thereby indicating that this may be potentially applied to the field.

The identification of gene locations that control favorable phenotypes is a critical step in plant breeding and gene cloning, where tightly linked markers can be used in marker-assisted selection during plant breeding. Traditional gene mapping is labor-intensive, time-consuming, and costly. Numerous DNA markers have been used to detect polymorphic sites between parents or DNA pools such as those identified by bulked-segregant analysis (BSA). Parts of these markers are polymorphic and only a few of these are linked to target genes. However, polymorphisms can readily be identified using next-generation sequencing (NGS). BSA has been recently combined with NGS technologies for gene mapping. Using these methodologies, we located *ty-5* within a 970-kb interval on chromosome 4 of tomato. SNP indices and analysis of ED values among polymorphic sites within this interval have allowed us to easily identify linkage markers for *ty-5*, which include CAPS, INDELs, and SNPs that can be subsequently used for further investigations.

Compared to the susceptible control line, Money maker, plants carrying TYLCV resistance genes still show low virus levels, but do not exhibit severe symptoms after inoculation with TYLCV. TYLCV can replicate and spread across tomato lines, regardless of the presence of resistance genes in the lines. Tomato lines with ‘resistance’ genes are thus not technically resistant, but rather tolerate low levels of TYLCV. We have thus named these genes based on the phenotype of each tomato line after inoculation with TYLCV. Genes that are associated with TYLCV susceptibility have thus been referred to as ‘S-genes’^23^. The discovery and utilization of S-genes is a novel strategy for disease resistance breeding.

In this study, a recessive gene in AVTO1227, namely, *ty-5*, was identified within a 14.5-kb interval on chromosome 4, where only a single gene, *pelota*, is located. Pelota has been demonstrated as a surveillance factor in the dissociation process of ribosomes into subunits^24,25^. TYLCV resistance genes differ from typical resistance genes, and of these, *ty-5* is the only one that was associated with recessive resistance. Two other examples of recessive resistance genes, *ol-2* and *pot-1*, have been reported in powdery mildew fungus and potyviruses, respectively^26,27^. These recessive resistance genes play key roles in pathogenesis, and natural or TILLING mutations involving these genes can result in broad-spectrum resistance in tomato. Resequencing of *ty-5* and its allele, *Ty-5*, demonstrated that there are two nucleotide transversions within the promoter region and one nucleotide transversion in an exonic region. The expression of these alleles did not change regardless of TYLCV inoculation. Consequently, variations in the exon may lead to protein changes. Considering that *ty-5* is a recessive resistance gene similar to ol-2 and pot-1, we hypothesize that *Ty-5* may be an S-gene that is important to TYLCV invasion. CRISPR/Cas9 methods may also be employed to edit the *Ty-5* allele of the Money maker tomato line.

Two different polymorphic regions were identified between *ty-5* and its susceptibility allele, *Ty-5*, in AVTO1227. Two transversions were found within the promoter of the *ty-5* gene, whereas a SNP was detected in the exon of *ty-5*. No difference in expression of *ty-5* or *Ty-5* was detected among all samples, regardless of whether the sample was inoculated with TYCLV. Thus, the two transversions in the *ty-5* promoter may not contribute to TYLCV resistance in AVTO1227. Future investigations should use the SNP within the exon to generate an amino acid substitution in *ty-5* of AVTO1227. The *pelota* gene, which is located within the *ty-5* region, has been demonstrated as a messenger RNA surveillance factor that plays a role in the dissociation of ribosomes into subunits when ribosomes are stalled. However, there is no current evidence that *pelota* participates in TYLCV resistance. TYLCV have six partially overlapping open reading frames, and similar to most viruses, TYLCV relies on the host cell machinery to complete their infection cycle. Consequently, investigations involving mutations in the *pelota* gene may elucidate its role in viral protein biosynthesis.

## Methods

### Plant materials and growth conditions

The TYLCV resistance gene accessions AVTO1227 and CLN2777A that carry the *ty-5* and *Ty-2* genotypes, respectively, were obtained from the Asian Vegetable Research and Development Center. The Money maker accession is susceptible to TYCLV. A cross between AVTO1227 (P1) and Money maker (P2) were performed, and the resulting F_1_ population was used as the female parent in generating the BC_1_P_1_ and BC_1_P_2_ lines. The F_1_ plants were then self-pollinated to obtain the F_2_ populations. Lastly, the F_2_ individuals were self-pollinated to generate F_2:3_ lines. The TYLCV resistant inbred line CLN2777A and the TYLCV susceptible line 9210 were used as controls to assess the success of TYLCV inoculation. All plants were grown at 26 °C under the same conditions.

### Whitefly reproduction and TYLCV inoculation

Whitefly feeding and reproduction was conducted in a phytotron. Six-week-old 9210 line seedlings were inoculated with whiteflies viruliferous for the TYLCV-IL strain. Whitefly-mediated TYLCV inoculation was considered successful when the disease index of the seedlings reached >55%. Disease evaluation was conducted when the abundance of viruliferous whitefly was high enough for inoculation. Three leaf-stage seedlings were used to evaluate disease states. Trays with seedlings were moved to the phytotron, and viruliferous whiteflies on line 9210 were introduced to the inoculated seedlings, followed by shaking of the seedlings three times each day to achieve uniform inoculation. Seven days after inoculation, the trays were moved to another phytotron. Whiteflies were killed using imidacloprid, and the seedlings were transplanted to the glasshouse. One single seedling was conducted per pot plant, as previously described^28^.

Agroinfiltration was used in RT-PCR analysis. An infectious TYLCV clone (kindly provided by Dr. Baolong Zhang, Jiangsu Academy of Agricultural Science, Nanjing, Jiangsu, China) was transformed to *Agrobacterium tumefaciens* strain LBA4404 and used to agroinoculate the tomato seedlings. *A. tumefaciens* containing the TYLCV clone at an OD_600_ of 0.5 was used in the inoculations. The leaves of three-week-old seedlings were infiltrated by pressure inoculation using a needleless syringe.

### Disease evaluation

Forty days after inoculation, the plants were assessed for TYLCV infection severity. The index of disease severity ranged from 0 to 4, where 0 = no visible symptoms and inoculated plants were similar to non-inoculated plants; 1 = mild yellowing of leaves at the apical point and no curly leaves; 2 = some yellowing and curling of apical leaves; 3 = strong yellowing and curling across a wide area of the inoculated plant, and slowed, but not arrested, growth; 4 = severe stunting, yellowing and curling, and plant growth ceased. Intermediate scores (e.g., 0.5 and 1.5) represent intermediate disease morphologies based on the above scale and previously described methods^29^.

### DNA extraction and genome pool construction

DNA was extracted using a modified CTAB technique, as described elsewhere^30^, and DNA concentrations were quantified with an Eppendorf BioSpectrometer machine (Eppendorf, Germany). Two genomic DNA pools were constructed for BSA-seq analysis, namely, the R-pool (resistant to TYLCV) and the S-pool (susceptible to TYLCV). Pools were constructed by mixing an equal concentration of DNA from 27 TYLCV resistant (grade = 0) and 27 TYLCV susceptible (grade = 4) F_2_ individuals.

### Whole genome resequencing data analysis

Illumina libraries for the R- and S-pools were prepared according to the manufacturer’s protocols. Libraries with mixtures of 300–500 bp DNA fragments were constructed following fragmentation, adapter ligation, size selection, and PCR enrichment. Paired-end sequencing of fragments was performed using the Illumina High-seq 2500 sequencing platform at Berry Genomics Co., Ltd. To generate high-quality clean reads, raw sequence reads were filtered and trimmed using the following criteria: reads that matched a minimum of 25 nt of the adaptor sequence on the 5′ end were trimmed; reads with >10% unknown nucleotides or ambiguous bases were removed; and reads in which the percentage of low-quality bases (base quality ≤3) was ≥50% were removed. The clean reads from both pools were aligned to the Heinz 1706 reference genome using BWA and SAMTools software. Duplicate removal was conducted using the Picard software. SNPs and INDELs were then assessed^22,31^.

### Gene location association analysis

A SNP index was used to indicate the proportion of reads harboring SNPs that differed from reference sequences. An Euclidean distance value (ED) was calculated by comparing SNPs across the two DNA genome pools as follows: SNP-index_alt_ = N_alt_/(N_alt_ + N_ref_), Δ(SNP − index_alt_) = SNP − index_alt_ (R-pool) − SNP − index_alt_ (S-pool), SNP-index_ref_ = N_ref_/(N_alt_ + N_ref_), Δ(SNP − index_ref_) = SNP − index_ref_ (R-pool) − SNP − index_ref_ (S-pool), ED = [Δ(SNP − index_ref_)^2^ + Δ(SNP − index_alt_)^2^]^1/2^. N_alt_ indicates the number of short reads that differed from the reference sequence. N_ref_ indicates the number of short reads that were identical to reference sequences. Using these formulae, we then assessed whether the measured values fell within the following ranges, −1 ≤ Δ(SNP − index) ≤ 1, 0 ≤ ED ≤ 1.414. An ED of 1.414 would indicate that all of the SNP-containing reads from one DNA genome pool differ from the reference sequences, and that the SNP sites from the other pool do not differ from the reference sequences. When the ED is near 0, the proportion of reads with SNPs is the same in both the R- and S-pools. Loess regression fitting was then used to determine the association and location of the resistance gene, based on previously described methods^21^. Genomic regions with values over the threshold were considered candidate regions that were associated with *ty-5*. The ED of INDEL sites was calculated using the INDEL-index and Δ(INDEL - index) as described above for calculating the ED for SNP regions. Sliding window analysis was applied to ED plots using 2-Mb window sizes and in 10-kb increments. The average ED of the SNPs in each window was then calculated as previously described^22,32^.

### Development of molecular markers for linkage map construction

Polymorphic SNP and INDEL sites among candidate *ty-5* regions were used to design molecular markers. Primers for each polymorphic site were designed using the Primer Premier 5.0 software. Endonuclease enzymes were used when SNPs could be used as cleaved amplified polymorphic sequence (CAPSA) markers. Alternatively, if no endonuclease was suitable for the SNP site, then PCR reaction products were amplified and sequenced to directly identify SNPs. CAPS and INDEL markers were detected on 2% agarose and 6% polyacrylamide gels. The PCR and enzyme digestion reactions were as described by Wang *et al.* 2012^30^. The genetic linkage map was constructed using the JOINMAP4.0 software and a LOD threshold of 3.0^33^.

### Expression analysis and *ty-5* sequence comparisons

Candidate gene expression experiments were conducted after genetic linkage mapping. Leaf samples were collected from 10 individuals of both AVTO1227 and Money maker lines before inoculation and seven days after inoculation. Three biological replicates were conducted for each sample. Total leaf RNA was extracted using the RNA simple Total RNA Kit (TIANGEN, Beijing, China). Reverse transcription of RNA to cDNA was conducted using the PrimeScript 1st Strand cDNA synthesis kit (TaKaRa, Japan). Primers for quantitative RT-PCR of *ty-5* were also designed using the Primer Premier 5.0 software. Primer specificity was evaluated by BLAST searches against the NCBI database and also via melt curve analysis after qPCR amplification. PCR amplifications were performed in the QuantStudio 6 Flex real-time thermal cycler (Thermo Fisher Scientific, USA) with 20-μL final reaction volumes containing 2.0 μL of cDNA, 0.4 μL of each primer (10 μM), 6.8 μL of sterile water, 0.4 μL of ROX Reference Dye II, and 10 μL (2×) SYBR Premix ExTaq™ II Kit (TaKaRa, Japan). The conditions for amplification were as follows: denaturation at 95 °C for 30 s, followed by 40 cycles of 95 °C for 5 s and 60 °C for 34 s. Expression levels of the selected genes were normalized to *GAPDH* expression levels. Three technical replicates of each sample were performed for *ty-5* and *GAPDH* expression. Relative gene expression was calculated using the 2^−ΔΔCT^ method^34^.

### Accession codes

The sequencing data of this study has been deposited in the NCBI Sequence Read Archive under the accession number PRJNA312569.
