# Author Correction: Application of Whole Genome Resequencing in Mapping of a Tomato Yellow Leaf Curl Virus Resistance Gene

**DOI:** 10.1038/s41598-021-86636-x

**Published:** 2021-03-19

**Authors:** Yinlei Wang, Jing Jiang, Liping Zhao, Rong Zhou, Wengui Yu, Tongmin Zhao

**Affiliations:** 1 Institute of Vegetable Crop, Jiangsu Academy of Agricultural Science, Nanjing, Jiangsu China; 2 Laboratory for Genetic Improvement of High Efficiency Horticultural Crops in Jiangsu Province, Nanjing, Jiangsu China

Correction to: *Scientific Reports* 10.1038/s41598-018-27925-w, published online 25 June 2018

Our Article describes ty-5 as a new gene, but it came to our attention after publication that the gene had been cloned in previous work^1^. Although our research ultimately identified the same gene, there are significant differences in materials, methods, and experimental design. We also compared the TYLCV content in different tomato lines, and we reported polymorphism information for 12 chromosomes from whole genome resequencing that can be used not only for ty-5 mapping, but also in other research. With this correction we give due credit to the Lapidot et al*.* paper and clarify the original contribution made by our paper.
